# American Medical Association, at Nashville

**Published:** 1857-07

**Authors:** 


### PROCEEDINGS OF MEDICAL SOCIETIES.

*Proceedings of the American Medical Association, held at Nashville, May* 5, 6, *and* 7, 1857.

The delegates and members assembled in the State Capitol, and were called to order by the President, Dr. Zina Pitcher, of Michigan.

Dr. C. K. Winston, chairman of the Committee of Arrangements, extended to the members, in behalf of the profession and citizens of Nashville, a cordial and appropriate welcome.

On calling the roll, one hundred and forty-six members answered to their names, as follows: Connecticut, 1; New Hampshire, 1; New York, 4; Pennsylvania, 4; Georgia, 11; Alabama, 11; Tennessee, 57; Louisiana, 4; Kentucky, 13; Indiana, 5; Illinois, 6; Missouri, 4; Michigan, 9; Iowa, 4; Ohio, 4; Wisconsin, 2; South Carolina, 4; Mississippi, 1; Arkansas, 1.

The names of the officers elected for the ensuing year were given in the June number of our Journal.

The Association continued in session three days, during which reports were presented on the following subjects: On Medical Topography and Epidemics, by Dr. Peregrine Wroth, of Maryland, and Dr. J. F. Posey, of Georgia.

Medical Topography and Fauna of Washington Territory, by Dr. G. Suckley, of the U. S. Army.

Medical Statistics of Washington Territory, by Dr. George Suckley, of U. S. Army.

Medical Flora of Washington and Oregon Territories, by Dr. G. A. Cooper.

A new Principle of Diagnosis in Dislocations of the Shoulder-joint, by Dr. L. A. Dugas, of Georgia.

Causes of Infant Mortality, etc., by Dr. D. Meredith Reese, of New York.

Blending and Conversion of the Types of Fever, by Dr. C. G. Pease, of Wisconsin.

Use of Cinchona in Malarious Diseases, by Dr. F. Trinkle, of Pennsylvania.

Nervous System in Febrile Diseases, by Dr. H. F. Campbell, of Georgia.

Ætiology and Pathology of Epidemic Cholera, by T. W. Gordon, of Ohio.

The foregoing reports and papers were all referred to the Committee on Publication, and they will doubtless constitute the contents of the next volume of Transactions of the Association. Dr. Boling, from the Committee on Prize Essays, reported that only four papers had been received, from which the two following were selected for premiums, viz.: 1st. On the Excreto-secretory System of Nerves, and its Relations to Physiology and Pathology, by Dr. H. F. Campbell, of Georgia; 2d. Experimental Researches Relative to the Nutritive Value and Physiological Effects of Albumen, Starch, and Gum, when singly and exclusively used for Food, by Dr. W. A. Hammond, assistant surgeon in the U. S. Army.

The only subject of general interest, which elicited much discussion, led to the adoption of the following resolutions, offered by Dr. Curry, of Tennessee, viz.:

*Whereas*, The subject of medical education has been committed at each annual session to standing committees, and various suggestions have been proposed which the Association has adopted, and recommended to private instructors and to the Medical Colleges,

*Resolved*, That a committee of five be appointed by the Committee on Nominations, as a special committee, to be composed of members who are in no respect connected with any medical school, to devise *a system of medical instruction*, to be presented for the consideration of this Association at its annual session in 1858.

*Resolved*, That the proposed system shall set forth a uniform basis upon which our medical institutions shall be organized, as well as have reference to the best mode of securing the preparatory medical instruction to the student; and that, consequently, the legitimate subjects to be embraced in said system will include primary medical schools, the number of professorships in medical colleges, the length and number of terms during the year, the requisite qualifications for graduation, and such other subjects of a general character as to give uniformity to our medical system, and preserve harmony and friendly intercourse in the ranks of the profession.

*Resolved*, That, upon the adoption of the proposed system by the Association, all institutions which may conform to it shall be entitled to representation at the annual sessions of this Association, and none others.

The committee called for by the first of these resolutions consists of Drs. James R. Wood and John Watson, of New York; R. La Roche, of Philadelphia; George R. Grant, of Memphis; and C. B. Nottingham, of Macon.

This is a good committee, and we hope they will give the subject that full and definite consideration which its importance demands.

The standing and special committees appointed to report at the next meeting of the Association are as follows:

STANDING COMMITTEES.

*Committee of Publication.*—Francis G. Smith, of Philadelphia, Chairman; Caspar Wister, of Philadelphia; R. C. Foster, of Nashville; A. J. Semmes, of Washington City; Samuel L. Hollingsworth, of Philadelphia; Samuel Lewis, of Philadelphia; H. E. Askew, of Delaware.

*Committee on Prize Essays.*—Grafton Tyler, of Georgetown, D. C., Chairman; J. C. Hall, of D. C.; J. F. May, of D. C.; Thomas Miller, of D. C.; A. J. Semmes, of D. C.; Joshua Riley, of D. C.; W. J. C. Duhamel.

*Committee of Arrangements.*—Harvey Lindsley, Chairman; W. J. C. Duhamel, Cornelius Boyle, P. H. Coolidge, G. M. Dove, A. Y. P. Garnett, Wm. P. Johnston, of D. C.

*Committee on Medical Education.*—G. W. Norris, of Philadelphia, Chairman; A. H. Luce, of Illinois; E. R. Henderson, of South Carolina; G. R. Grant, of Tennessee; T. S. Powell, of Georgia.

*Committee on Medical Literature.*—A. B. Palmer, of Detroit, Chairman; A. F. Alexander, of Alabama; J. M. Mosgrove, of Ohio; P. Cassidy, of Pennsylvania; S. Pollok, of Missouri.

*Vacancies in Committee on Medical Topography and Epidemics.*—T. B. Shuford, to fill the vacancy caused by the death of Dr. Grafton, of Mississippi; C. W. Parsons, to fill the vacancy caused by the resignation of Joseph Mauran, of Rhode Island.

SPECIAL COMMITTEES.

*Spontaneous Umbilical Hemorrhage of the Newly Born.*—J. Foster Jenkins, of New York.

*Influence of Marriages of Consanguinity upon Offspring.*—Dr. Bemiss, of Ky.

*Functions of different portions of the Cerebellum.*—E. Andrews, of Ill.

*Causes of the Impulse of the Heart and the Agencies which influence it in Health and Disease.*—J. W. Corson, of New York City.

*Treatment of the Results of Obstructed Labor.*—J. Marion Sims, of N. Y.

*Treatment best adapted to each variety of Cataract, with the Method of Operation, Place of Election, Time, Age, etc.*—Mark Stephenson, of N. Y.

*Human, Animal, and Vegetable Parasites.*—Joseph Leidy, of Philadelphia.

*Best Substitutes for Cinchona and its Preparations in the Treatment of Intermittent Fever, etc.*—B. S. Woodward, of Indiana.

*Intimate Structure and Pathology of the Kidney.*—Charles E. Isaacs, of N. Y.

*Etiology and Pathology of Epidemic Cholera.*—T. W. Gordon, Georgetown, Brown county, Ohio.

*Inflammation of the Cervix Uteri.*—Henry H. Miller, of Louisville, Ky.

*On Milk Sickness.*—W. H. Byford.

*Best Means of Causing an Increase of the number of Essays.*—Drs. Leidy, Wood, and Meigs, of Pa.

*Changes produced in Composition and Properties of Milk.*—N. S. Davis, of Ill.

*Stomatitis Materna.*—Dr. C. McGugin, of Iowa.

*On Criminal Abortion, with a view to its general suppression.*—H. N. Storer, of Boston.

The committee recommended the amendment of the third article of the constitution, in relation to *meetings*, by inserting after the words, "first Tuesday in May," the words, "*or the first Tuesday in June*," and also, by inserting after the words, "shall be determined," the words, "*with the time of meeting*."

*Special Committee on the present state of Science as regards the Pathology and Therapeutics of the Reproductive Organs of the Female.*—Dr. Fordyce Barker, of N. Y.

*On Moral Insanity.*—D. Meredith Reese, M. D., of New York.

*On Calculi and Diseases of the Urinary Organs in Iowa, Minnesota, and Nebraska.*—Dr. J. C. Hughes, of Keokuk.

*On the Nature, Tendency, and General Treatment of Syphilitic Buboes.*—Moses Gunn, of Detroit.

